# Prevalence of the Brazilian *TP53* Founder c.1010G>A (p.Arg337His) in Lung Adenocarcinoma: Is Genotyping Warranted in All Brazilian Patients?

**DOI:** 10.3389/fgene.2021.606537

**Published:** 2021-02-02

**Authors:** Igor Araujo Vieira, Tiago Finger Andreis, Bruna Vieira Fernandes, Maria Isabel Achatz, Gabriel S. Macedo, Daniel Schramek, Patricia Ashton-Prolla

**Affiliations:** ^1^Programa de Pós-Graduação em Genética e Biologia Molecular, Universidade Federal do Rio Grande do Sul, Porto Alegre, Brazil; ^2^Laboratório de Medicina Genômica, Centro de Pesquisa Experimental, Hospital de Clínicas de Porto Alegre, Porto Alegre, Brazil; ^3^Curso de Graduação em Biomedicina, Universidade Federal de Ciências da Saúde de Porto Alegre, Porto Alegre, Brazil; ^4^Departamento de Oncogenética, Hospital Sírio Libanês, São Paulo, Brazil; ^5^Programa de Medicina Personalizada, Hospital de Clínicas de Porto Alegre, Porto Alegre, Brazil; ^6^Centre for Molecular and Systems Biology, Lunenfeld-Tanenbaum Research Institute, Mount Sinai Hospital, Toronto, ON, Canada; ^7^Department of Molecular Genetics, University of Toronto, Toronto, ON, Canada

**Keywords:** *TP53* gene, p53 protein, lung adenocarcinoma, founder variant, *TP53* (p.Arg337His), R337H, non-small cell lung cancer, Li-Fraumeni syndrome

## Abstract

In Southern and Southeastern Brazil, there is a germline pathogenic variant with incomplete penetrance located in the oligomerization domain of *TP53*, c.1010G>A (p.Arg337His). Due to a founder effect, the variant is present in 0.3% of the general population of the region. Recently, this variant was identified in 4.4 and 8.9% of two apparently unselected, single center case series of Brazilian lung adenocarcinoma (LUAD) patients from the Southeastern and Central regions of the country, respectively. In the present study, our aim was to examine *TP53* c.1010G>A allele and genotype frequencies in LUAD samples obtained from patients diagnosed in Southern Brazil. A total of 586 LUAD samples (tumor DNA) recruited from multiple centers in the region were tested, and the mutant allele was identified using TaqMan^®^ assays in seven cases (7/586, 1.2%) which were submitted to next generation sequencing analyses for confirmation. Somatic *EGFR* mutations were more frequent in *TP53* c.1010G>A carriers than in non-carriers (57.1 vs. 17.6%, respectively). Further studies are needed to confirm if *TP53* c.1010G>A is a driver in LUAD carcinogenesis and to verify if there is a combined effect of *EGFR* and germline *TP53* c.1010G>A. Although variant frequency was higher than observed in the general population, it is less than previously reported in LUAD patients from other Brazilian regions. Additional data, producing regional allele frequency information in larger series of patients and including cost-effectiveness analyses, are necessary to determine if *TP53* c.1010G>A screening in all Brazilian LUAD patients is justified.

## Introduction

In Southern and Southeastern Brazil, a germline founder pathogenic variant with incomplete penetrance, c.1010G>A (rs121912664), also known as R337H or p.(Arg337His) has been detected in 0.3% of the general population (Achatz et al., 2009; Custódio et al., 2013). It is located in the oligomerization domain (exon 10) of *TP53*, and it is associated with Li-Fraumeni syndrome (LFS). Carriers are at a high risk for developing a wide spectrum of tumors and are prone to develop multiple primary cancers at different ages. The core tumors in LFS patients include early onset breast cancer, soft-tissue sarcomas, brain tumors and adrenocortical carcinomas (Achatz et al., 2007; Giacomazzi et al., 2013; Achatz and Zambetti, 2016). Lung cancer (LC), especially lung adenocarcinoma (LUAD) of the lepidic subtype, has been reported in LFS families and is included in the Chompret criteria for genetic testing of suspected patients, but there is limited evidence for a strong association of its occurrence with germline *TP53* variants (Tinat et al., 2009; Ricordel et al., 2015; Caron et al., 2017). Although *TP53* is considered one of the most commonly mutated genes in solid tumors, somatic occurrence of *TP53* c.1010G>A is extremely rare. In the IARC *TP53* database [International Agency for Research on Cancer TP53 database (Iarc), 2020], c.1010G>A has been described in only 4 of 28,869 solid tumors. In other public databases, its frequency in solid tumors is also very low. In COSMIC [Catalogue Of Somatic Mutations In Cancer (Cosmic), 2020], for instance, among over 20.000 samples, *TP53* c.1010G>A is not present, although G > T and G > C alterations in codon 1,010 do occur. In contrast, two lung tumors harboring germline c.1010G>A have been described in the IARC *TP53* database. Finally, Nogueira et al. (2015) reported a mixed acinar/bronchiolo-alveolar carcinoma in a known germline carrier of the *TP53* c.1010G>A variant (Nogueira et al., 2015).

LC is the leading cause of cancer related deaths worldwide, responsible for 1.7 million deaths every year. In Brazil, the National Cancer Institute [Instituto Nacional de Câncer José Alencar Gomes da Silva (Inca), 2020] estimated 30,200 new LC cases in 2020, rendering it the second most common solid tumor in the country. In Southern Brazil, LC is the third most common cancer diagnosed in adults, with non-small cell lung cancer (NSCLC) accounting for approximately 85% of all LCs cases. Recently, Couto et al. (2017) genotyped *TP53* c.1010G>A in 45 NSCLC patients from a single center in the central region of Brazil, where this variant has not been explored and its population prevalence has not been determined. They identified 4 (8.9%) heterozygotes, a surprisingly high variant frequency for a small, apparently unselected cohort (Couto et al., 2017). Importantly, a more recent single center study in Southeastern Brazil also observed a high prevalence of *TP53* c.1010G>A in an unselected series of 114 *EGFR*-positive LUAD patients: the variant was present in 4.4 and 12.5% of samples when considering diagnosis at any age or before the age of 50 years, respectively (Barbosa et al., 2020). Furthermore, the authors assessed LUAD tumors diagnosed in known *TP53* c.1010G>A carriers and observed that LUAD tumors from 8/9 (89%) *TP53* c.1010G>A carriers harbored an activating *EGFR* variant. To our knowledge, the *TP53* c.1010G>A variant has not been described in other studies assessing somatic *TP53* variants in sporadic lung adenocarcinoma (LUAD) patients (Greenman et al., 2007; Ding et al., 2008; Hammerman et al., 2012; Imielinski et al., 2012; Peifer et al., 2012; Rizvi et al., 2015; Jordan et al., 2017). Thus, in the present study, our goal was to examine the *TP53* c.1010G>A allele and genotype frequencies in a series of 586 LUAD samples obtained from patients diagnosed in multiple centers of the three states of Southern Brazil, a region with the highest population frequency of this particular variant observed to date, regardless of their clinical features.

## Methods

### Study Subjects and Ethical Aspects

A total of 586 LC samples derived from a cohort described in a previous study from our group (Andreis et al., 2019) were analyzed. Patients were originally recruited for somatic mutation testing in *EGFR* (exons 18–21), *KRAS* (exons 2 and 3), *BRAF* (exons 11 and 15), and *NRAS* (exons 2 and 3) genes from different hospitals and clinics distributed in 22 healthcare centers located in the three states of the southern region of Brazil: Rio Grande do Sul (*N* = 496), Santa Catarina (*N* = 20), and Paraná (*N* = 70). Patients were not selected on previous cancer history or family history of cancer. Pathologic analyses confirmed typical adenocarcinoma histology in all cases. However, histological subtype data were available for only a small proportion (72/586) of patients. The mutation status of *EGFR*, *BRAF*, *KRAS*, and *NRAS* genes was evaluated using technical procedures as previously reported (Andreis et al., 2019). Genotyping was performed in a central laboratory, a diagnostic precision medicine program established in a tertiary care hospital in southern Brazil. Before initiation of this study, age at LUAD diagnosis, *EGFR*/*KRAS/BRAF* status, and histological subtype (when available) were annotated and samples were posteriorly de-identified. A consent waiver was approved by the Institutional Review Board specifically for the *TP53* c.1010G>A analysis, given that patient identification using only age at diagnosis, *EGFR*/*KRAS*/*BRAF*/*TP53* status and LUAD histological subtype would not allow patient re-identification.

### DNA Extraction and *TP53* c.1010G>A Genotyping

Tumor DNA was extracted using the ReliaPrep^TM^ FFPE gDNA Miniprep System (Promega), according to the manufacturer’s recommendations. Next, TaqMan^®^ allelic discrimination analyses of the pathogenic variant *TP53* c.1010G>A (rs121912664) were performed according to Applied Biosystems^®^ standard protocols (Applied Biosystems, Carlsbad, CA United States), using fluorescent allele-specific probes as previously published (Fitarelli-Kiehl et al., 2016). An attempt to confirm rs121912664-positive results identified by TaqMan^®^ by next generation sequencing (NGS) for determination of allele frequencies was made. Briefly, NGS of the *TP53* entire coding region (exons 2–11) and 70 pb exon-intron boundaries was done using a custom panel (Thermo Fisher Scientific, CA, United States, reference number TP53.20140108.designed) on the Ion Torrent-Personal Genome Machine (PGM) platform. Amplicon library was prepared using the Ion AmpliSeq^TM^ Library Kit 2.0 (Thermo Fisher Scientific, CA, United States). PCR products were then sequenced on the Ion GeneStudio S5 system (Ion Torrent Systems Inc., Gilford, NH, United States). NGS results were interpreted using the Ion Reporter software considering a minimum coverage of 100X by amplicon. Integrative Genomics Viewer (IGV) was used for visualization of the mapped reads. Human *TP53* cDNA sequence corresponding to the NM_000546.5 was used as a wild-type (WT) reference.

### Statistical Analyses

Genotype and allele frequencies were estimated by simple counting. Clinical and molecular features of LUAD patients were assessed using descriptive statistics. Considering the low number of mutant alleles found in our study and limitations in clinical data availability, it was not possible to perform any meaningful statistical test in our comparisons between groups of carriers and non-carriers (see more in Results section). SPSS^®^ version 18 (SPSS^®^ Inc., Chicago, IL, United States) was used for data handling and for all descriptive analyses.

## Results

A total of 586 LUAD samples were included in this study. Clinical and molecular data are summarized in Table 1. Histopathological subclassifications were available only for 72 cases (12.3%). The most common subtypes were acinar (65.3%) and lepidic (34.7%). Moreover, the majority of LUAD samples (502/586, 85.7%) were tested for somatic mutations in *EGFR, KRAS, BRAF* and *NRAS* driver genes. Somatic mutations were mostly identified in *KRAS* (29.5%) and *EGFR* (21.1%).

**TABLE 1:** Clinical and molecular features of 586 patients with lung adenocarcinoma (LUAD) included in this study.

**Features**	Median (IR)*	***N* (%)^†^**
Age at tumor diagnosis, years	67 (16)	–
Gender		
Male		271 (46.2)
Female		315 (53.8)
Histological subtype		72 (12.3)
Acinar		47 (65.3)
Lepidic		25 (34.7)
Patients tested for somatic alterations^‡^		502 (85.7)^‡^
*EGFR* mutation		106 (21.1)
*KRAS* mutation		148 (29.5)
*BRAF* mutation		
None		233 (46.4)
Carriers of *TP53* c.1010G>A (p.Arg337His)^§^		7 (1.2)^§^

*IR, interquartile range.

*^†^The percentage was calculated over the total number of genotyped samples (586) and over the number of cases for which the specified clinical data was available.*

*^‡^Tested for somatic *EGFR*, *KRAS*, *BRAF*, and *NRAS* mutations. Specific regions evaluated in each gene are detailed in the “Materials and Methods.*

*”^§^ Seven carriers were identified using TaqMan^®^ assays, but heterozygous genotype was confirmed by NGS in only five positive cases due to limitations in sample availability for the remaining two patients.*

Specific *TP53* c.1010G>A (p.Arg337His) genotyping by TaqMan^®^ resulted in the identification of seven heterozygotes (GA genotype). Heterozygous genotype and mutant allele (A) frequencies were thus defined at 7/586 (1.2%) and 7/1,172 (0.6%), respectively. All patients in this subgroup were diagnosed with LUAD after age 50 years, and the median age at tumor onset was 60 years. In addition, most c.1010G>A tumors also had somatic *EGFR* (4/7, 57.1%) variants and none of them had a *KRAS* variant. Importantly, heterozygous genotype was confirmed by NGS in five (p.Arg337His)-positive cases (5/586, 0.85%). In the other two positive samples, further analyses were not possible due limitations in sample availability. Mutant allele frequencies determined by NGS were close to 50% in 3/5 cases, suggesting that at least in these cases, the variant may also be present in the germline. Details on each of the heterozygous samples are summarized in Table 2. Additional *TP53* alterations detected in the tumors by NGS are depicted in **Supplementary Table 1**.

**TABLE 2:** Characterization of LUAD tumors with an identifiable *TP53* c.1010G>A pathogenic variant.

**Identifier**	**Gender**	**Age at LUAD diagnosis (years)**	**Histological subtype**	**Somatic *EGFR* mutation**	***TP53* c.1010G>A zygosity**	WT*/*TP53* c.1010G>A allele frequency (coverage)	**Percentage of tumor cells in the sample**
1	Male	57	NA^†^	p.(Leu858Arg)	Heterozygous	0.52/0.48 (2,400x)	40%
2	Male	65	Lepidic	p.(Ser768_Asp770dup)	Heterozygous	0.37/0.63 (2,259x)	30%
3	Female	55	NA	p.(Leu858Arg)	Heterozygous	0.24/0.76 (4,000x)	60%
4	Female	60	Lepidic	None	Heterozygous	0.42/0.58 (1,802x)	40%
5	Female	74	NA	p.(Leu858Arg)	Heterozygous	0.42/0.58 (1,835x)	70%
6	Female	54	Lepidic	Inconclusive^§^	Heterozygous	NP^‡^	5%
7	Female	62	Acinar	Inconclusive	Heterozygous	NP	40%

* WT, wild-type allele; ^†^ NA, not available; ^‡^ NP, not performed; ^§^ Inconclusive status due to technical limitations, such as availability of a low concentration of DNA extracted from the tumor tissue, and/or poor quality/purity of the tumor DNA, leading to inadequate results (low coverage) in the NGS analysis.

Lastly, a comparison of the available clinical and molecular features between *TP53* c.1010G>A carriers identified by TaqMan^®^ and non-carriers is presented in Table 3 and **Supplementary Figure 1**. A difference between groups was observed for median age at cancer diagnosis and histological subtype but the number of mutant allele carriers (7/586) was too small to make meaningful comparisons. LUAD from three carriers were of the lepidic subtype (3/7, 42.8%), however, it is important to emphasize that three of the seven heterozygotes had no complete histological data, which hindered our ability to analyze a possible association between the c.1010G>A variant and occurrence of the lepidic subtype. Interestingly, the presence of somatic *EGFR* mutations was found in a much higher frequency in *TP53* c.1010G>A carriers than in non-carriers (57.1 vs. 17.6%, respectively).

**TABLE 3:** Comparison of clinical and molecular features between LUAD samples of *TP53* c.1010G>A carriers and non-carriers.

**Features**	**Carriers, *N* = 7**	**Non-carriers, *N* = 579**
Median age at diagnosis, years (IR)*	60 (10)	67 (16)
Histological subtype, *N* (%)	4 (57.1)	68 (11.7)
Acinar	1 (14.3)	46 (7.9)
Lepidic	3 (42.8)	22 (3.8)
Patients with other somatic alterations, *N* (%)^†^	5 (71.4)	497 (85.8)
*EGFR* mutation	4 (57.1)	102 (17.6)
*KRAS* mutation	0	148 (25.6)
*BRAF* mutation	0	15 (2.6)
Median age at diagnosis restricted to *EGFR*-mutant subtype, years (IR)	61 (16)	67.5 (20)
		

*IR, interquartile range.

*^†^A total of 502/586 LUAD cases were tested for somatic *EGFR*, *KRAS*, *BRAF*, and *NRAS* mutations.*

## Discussion

The *TP53* founder variant c.1010G>A, widely referred as R337H, is likely responsible for a significant proportion of the cancer burden in the Southern and Southeastern regions of Brazil due to its high frequency in the population (Achatz et al., 2009; Garritano et al., 2010). In a landmark study conducted in 171,000 newborns from the Southern Brazilian State of Paraná the variant was identified in 461 individuals (∼0.3%) (Custódio et al., 2013). Previous analyses performed by our research group (Palmero et al., 2008) identified a similar prevalence (2/750, ∼0,3%) in a cohort of healthy, asymptomatic women participating in a community-based breast cancer screening program in the state of Rio Grande do Sul (Southern Brazil). Furthermore, it has been well-documented that the variant exhibits incomplete, variable penetrance resulting in significant inter- and intra-familiar heterogeneity in phenotypic presentation with some carriers surviving without any cancer diagnosis to older ages and others having the typical LFS phenotype, with one or more cancers diagnosed in childhood and/or adult life (reviewed in Achatz and Zambetti, 2016). Unlike most *TP53* somatic and/or germline deleterious variants involved in tumorigenesis (i.e., those located in the DNA-binding domain of p53 protein), c.1010G>A is located in the p53 oligomerization domain and has been associated with a unique intracellular pH-dependent effect on protein stability, through which the mutant protein retains some partial tumor suppressor activity (DiGiammarino et al., 2002; Zerdoumi et al., 2017). More recently, a knock-in animal model containing the homologous *TP53* R337H variant (mouse R334H) demonstrated that this alteration triggers reduced formation of p53 dimers and tetramers (deficient oligomerization capacity) compared to WT *p53* in mouse liver tissues after exposure to a specific carcinogen (Park et al., 2018). Interestingly, the homozygous *p53* R334H mutant mice exposed to this carcinogen showed an increased liver tumor development, while mice with the same genotype that were not under exposure to the liver carcinogen developed normally without any significant difference in terms of either cancer incidence or life span compared with WT mice (Park et al., 2018, 2019).

Given its relevance, prevalence of the mutant allele (germline and/or somatic DNA analysis) has been extensively investigated in different Brazilian cohorts of patients with several tumor types, regardless of cancer family history (FH). In three studies evaluating patients with adrenocortical carcinoma (ACC), c.1010G>A was present in 90–97%, independent of cancer FH (Ribeiro et al., 2001; Seidinger et al., 2011; Mastellaro et al., 2018). Moreover, the variant was found in 69, 8.4, and 7.3% of unselected subjects diagnosed with choroid plexus carcinoma, neuroblastoma and osteosarcoma, respectively (Seidinger et al., 2011, 2015). Prevalence of the variant in breast cancer (BC) patients from southern and southeastern Brazil was also studied. In three independent case series, prevalences of 3.4% (familial BC but without a clear LFS phenotype) and 2.5–8.6% (BC patients unselected for cancer FH diagnosed in different age groups) were observed (Giacomazzi et al., 2014; Hahn et al., 2018).

In the present study, we identified seven LUAD tumors harboring the founder *TP53* variant among 586 samples recruited from patients in southern Brazil, a cohort described in detail elsewhere (Andreis et al., 2019). Remarkably, all variant-positive tumors were diagnosed over age 50 years. The late onset of LC in *TP53* c.1010G>A carriers is in agreement with a recent finding showing a lifelong cancer risk pattern characterized by a bimodal age distribution: one peak in the first 10 years of life associated mainly with ACC and CNS tumors, and a second peak in the fifth decade in which different tumor types occur, including LC (Mastellaro et al., 2017). Although LC (especially LUAD) is not among the core, most frequent tumors originally described in LFS, it has been described in Brazilian LFS families with the *TP53* c.1010G>A variant (Barbosa et al., 2020), together with other atypical tumors such as papillary thyroid and renal cancers (Achatz and Zambetti, 2016).

Although *TP53* c.1010G>A prevalence in LUAD reported in the present study (1.2%) is substantially higher than the population frequency observed in large cohorts of healthy individuals from the same Brazilian region (0.3%) (Palmero et al., 2008; Custódio et al., 2013), it is much lower than the prevalences previously described by Couto et al. (2017), and Barbosa et al. (2020). The conflicting results between our study and the study performed by Couto et al. (2017) may be explained by two main reasons: (a) small sample size along with a putative selection bias in patient recruitment in the study conducted by Couto et al. indirectly evidenced by high proportion of carriers identified with a cancer FH; and (b) employment of different variant screening approaches (PCR-RFLP vs. TaqMan^®^), since use of restriction endonucleases may be associated with increased false-positive rates (Uemura et al., 2004; Kang et al., 2009). The study by Barbosa et al. (2020), although performed on a larger sample size, is also a single center study developed in an institution that has been a reference center for the diagnosis and follow-up of LFS families. In addition, one cannot exclude presence of specific, regional environmental factors (carcinogenic exposure, similar to what has been observed in mouse models) which might act triggering LUAD carcinogenesis in c.1010G>A carriers and result in a variable susceptibility to LUAD in carriers from different geographic regions.

Another interesting finding of our study was the co-occurrence of *TP53* c.1010G>A and activating *EGFR* mutations, which is in agreement with two previous reports (Barbosa et al., 2020; Mezquita et al., 2020). Studying LFS patients with a germline *TP53* pathogenic variant (either DNA-binding variants or the founder c.1010G>A) and LUAD, Barbosa et al. (2020) and Mezquita et al. (2020) identified somatic *EGFR* alterations in 89 and 85% of the tumors, respectively. In our study, *EGFR* variants occurred in 57.1% of the *TP53* c.1010G>A-positive tumors, a lower proportion than reported before, but our cohort was not of LFS patients. In addition, when looking at 114 LUAD patients with *EGFR* mutations from a non-LFS cohort, Barbosa et al. (2020) observed a *TP53* codon 337 variant in 5.3%, which was further increased to 12.5% (4/32) when considering only patients diagnosed before age 50 years. In our study, *TP53* c.1010G>A was present in 4/106 (3.8%) LUAD samples with activating *EGFR* mutations, and all patients with both alterations were diagnosed after the age of 50 years. Importantly, ethnic ancestry has major impact on the incidence of *EGFR* mutation status in NSCLC patients, being lower (around 10%) in regions with higher European ancestry representation (Gahr et al., 2013). The prevalence of European ancestry in the population studied here, i.e., from Southern Brazil, has been estimated at 80–90%, the highest among all Brazilian regions (Ruiz-Linares et al., 2014). In agreement with this observation, several studies evaluating the human leukocyte antigen genotypic diversity in this region also confirmed a high European ancestry and a meaningful difference from Asians or even Brazilian indigenous populations (Castro et al., 2019).

**FIGURE 1:**
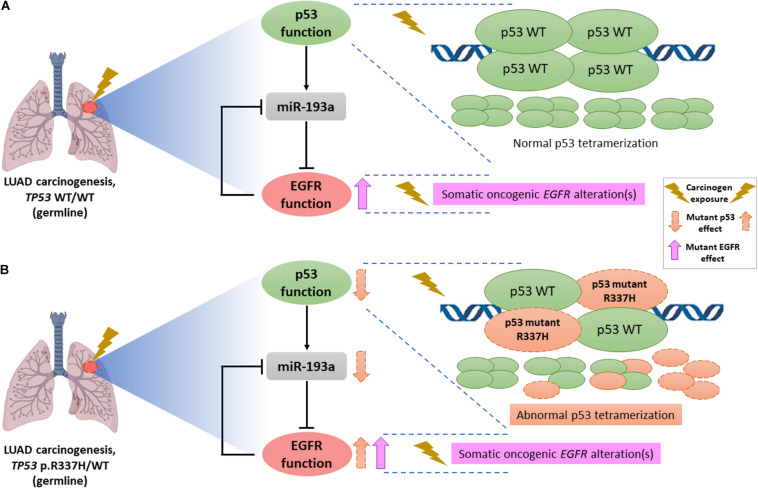
Schematic representation of the hypothetical interaction between mutant p53 (due to hypomorphic *TP53* germline variants such as c.1010G>A, p.R337H) and mutant EGFR (due to somatic activating *EGFR* alterations) in NSCLC tumors. **(A)** Lung carcinogenesis in an individual without germline *TP53* pathogenic variants, i.e., a homozygous context of wild-type (WT) p53 (*TP53* WT/WT genotype) characterized by constitutive formation of normal p53 tetramers in response to genotoxic stress and/or carcinogenic exposure. Occurrence of an activating *EGFR* somatic event leads to an EGFR upregulation. Moreover, according to previous functional studies, there is a link between p53 and EGFR through miR-193a, forming a double-negative feedback loop that contributes to NSCLC tumorigenesis. **(B)** LUAD formation process in a carrier of the germline *TP53* p.R337H (*TP53* p.R337H/WT genotype). To explain the frequent co-occurrence of germline *TP53* and somatic *EGFR* variants recently described in different case series, we postulate that the deficient p53 oligomerization capacity conferred by the *TP53*-p.R337H founder allele, specifically in the context of exposure to carcinogenic environmental factors triggers reduced tumor-suppressive p53 functions in lung tissue, representing an initial factor (first hit) in NSCLC/LUAD carcinogenesis. Subsequent mutational events at the somatic level (possibly resulting also from environmental exposure factors), including oncogenic *EGFR* variants (second hit) could dramatically impair the p53/miR-193a/EGFR feedback loop regulation, acting as a mechanism driving the NSCLC/LUAD development.

Indeed, the frequent co-occurrence of *TP53* and *EGFR* sequence variants is striking and it might be explained by a p53/miR-193a/EGFR feedback loop mechanism previously reported as a driving force for NSCLC tumorigenesis (Wang et al., 2019). *In vitro* and *in vivo* studies demonstrated that WT p53 directly activates miR-193a transcription and, in turn, miR-193a directly targets *EGFR*, whereas *EGFR* functions as a transcriptional repressor to negatively control miR-193a expression, forming a feedback loop (Figure 1A). Considering the repeated reports of co-occurrence of *TP53* and *EGFR* variants in LUAD, including the present study, we hypothesize that occurrence of an activating *EGFR* somatic event on a background of a tissue already harboring a mutant (germline) *TP53* allele (such as c.1010G>A) may result in an impairment of this loop function, promoting NSCLC formation and tumor growth (Figure 1B). In parallel, it has been widely described that, upon conditions of genotoxic stress in the cellular context, presence of functional *TP53* germline variants, especially hypomorphic variants (c.1010G>A), leads to a loss of p53 tumor-suppressive functions (such as DNA damage repair and cell-cycle arrest), triggering a genomic instability that, in turn, may promote the accumulation of somatic mutations at different genes (Zerdoumi et al., 2017; Park et al., 2018), which could include the hotspot regions of somatic *EGFR* mutations. Additionally, a previous study suggested that radiation-induced cancers are more common among LFS patients (harboring germline *TP53* pathogenic variants) (Heymann et al., 2010). Although the underlying mechanism is not known, in recent reports of *EGFR*-mutated lung tumors in LFS patients, researchers hypothesized that the first genetic hit was a germline *TP53* mutation and suggested that chemotherapy or radiotherapy (i.e., genotoxic stress-inducing conditions) promoted a second hit, which might have consisted specifically of somatic *EGFR* mutations (Michalarea et al., 2014; Ricordel et al., 2015). Further functional studies are required to confirm this latter hypothesis, as well as to elucidate, mechanistically, the reason why patients with *TP53* germline mutations seem to harbor, more frequently, activating *EGFR* mutations in lung tissue.

Finally, the results of the current study must be interpreted with caution, considering its limitations. First, LUAD specimens were obtained retrospectively from a diagnostic cohort de-identified for this study, hindering the inquiry about clinical data, such as histological subtype, smoking habit, cancer FH, and ethnic ancestry. Second, for the same reason, germline screening for the variant was not done and, thus, the study design employed here did not allow contact with R337H-positive patients or their relatives. Lastly, the limited availability of samples did not allow additional analyses of the tumor samples such as LOH testing.

In conclusion, when compared to previous studies in Brazilian patients with LUAD, the prevalence of *TP53* c.1010G>A, although higher than expected for the general population, was much lower in our series from Southern Brazil, suggesting that there may be regional variations. The variability observed so far, in the absence of large prevalence studies in different regions of the country, and also without a more detailed cost-effectiveness analysis, do not allow, in our view, proposition of a general recommendation of testing all Brazilian LUAD patients for *TP53* c.1010G>A. Further studies assessing presence of *TP53* germline variants, or at least the founder c.1010G>A variant, in Brazilian LUAD patients, regardless of the age at tumor diagnosis and especially if they harbor activating *EGFR* mutations, should be undertaken in order to determine if universal screening for *TP53* c.1010G>A is justified. Ultimately, predictive testing in healthy family members of variant-positive LUAD probands might be useful to assess the clinical actionability toward the occurrence of other tumor types, and future cost-effectiveness analysis should include this clinical actionability after predictive testing.

## Data Availability Statement

The raw data supporting the conclusions of this article will be made available by the authors, without undue reservation.

## Ethics Statement

The studies involving human participants were reviewed and approved by Institutional Review Board from Hospital de Clinicas de Porto Alegre (IRB-HCPA). Written informed consent for participation was not required for this study in accordance with the national legislation and the institutional requirements.

## Author Contributions

IV, TA, GM, and PA-P conceived the work and conception design of the brief research report. IV, TA, and PA-P designed the draft of the manuscript and carried out the statistical analyses. TA, GM, and PA-P were involved in recruitment of tumor DNA samples and retrospective search of clinical data. TA, BF, IV, GM, MA, and DS performed the genotyping and NGS analyses, as well as the interpretation of results from these experiments. IV, TA, MA, GM, DS, and PA-P helped to draft the manuscript. PA-P supervised the work. All authors revised the manuscript critically, contributed with interpretation of the findings and gave final approval of the version to be published.

## Conflict of Interest

The authors declare that the research was conducted in the absence of any commercial or financial relationships that could be construed as a potential conflict of interest.
